# Developmental trends in the facilitation of multisensory objects with distractors

**DOI:** 10.3389/fpsyg.2014.01559

**Published:** 2015-01-20

**Authors:** Harriet C. Downing, Ayla Barutchu, Sheila G. Crewther

**Affiliations:** ^1^School of Psychological Science, La Trobe UniversityMelbourne, VIC, Australia; ^2^Department of Experimental Psychology, University of OxfordOxford, UK

**Keywords:** multisensory, audiovisual, facilitation, distractors, development, variance, race model

## Abstract

Sensory integration and the ability to discriminate target objects from distractors are critical to survival, yet the developmental trajectories of these abilities are unknown. This study investigated developmental changes in 9- (*n* = 18) and 11-year-old (*n* = 20) children, adolescents (*n* = 19) and adults (*n* = 22) using an audiovisual object discrimination task with uni- and multisensory distractors. Reaction times (RTs) were slower with visual/audiovisual distractors, and although all groups demonstrated facilitation of multisensory RTs in these conditions, children's and adolescents' responses corresponded to fewer race model violations than adults', suggesting protracted maturation of multisensory processes. Multisensory facilitation could not be explained by changes in RT variability, suggesting that tests of race model violations may still have theoretical value at least for familiar multisensory stimuli.

## Introduction

Successful interaction with the environment requires individuals to discriminate relevant sensory signals from “noise.” To be safe, a child often needs to be able to rapidly identify his/her mother in a crowded shop or seek out a teammate in a schoolyard soccer game. Indeed multisensory information can enhance information processing in both adults and children (for reviews see e.g., Calvert et al., 2004; Bremner et al., 2012). However, the developmental trajectory of multisensory object processing in audiovisually distracting environments has not been reported, though it is of significant evolutionary relevance and has important implications for cognitive development (e.g., Johnson and Mareschal, 2001; Bremner and Spence, 2008; Barutchu et al., 2011).

In adults, the greatest multisensory benefits are observed for correlated multisensory stimuli that are in close spatial and temporal proximity (e.g., Parise et al., 2012; and see Stein and Meredith, 1993 for physiological theory from animal models). Processing of concurrent multisensory information has been reported to begin very early in life for human infants (e.g., Aldridge et al., 1999). Some aspects of multisensory processing speed and accuracy improve very early with age (e.g., Neil et al., 2006), while others remain immature throughout childhood across a variety of sensory modalities and tasks (e.g., Gori et al., 2008; Nardini et al., 2008, 2013; Barutchu et al., 2009; Maidment et al., 2014; Petrini et al., 2014; Baart et al., 2015). Stable adult levels of multisensory facilitation have been first reported in early adolescence (Brandwein et al., 2011), when multisensory reaction times (RTs) are reliably faster than would be predicted by parallel competing sensory systems (i.e., race models).

Developmental changes in multisensory processing may be modulated by changes in sensory dominance (Nava and Pavani, 2013). For example, the Colavita visual dominance effect, where adults typically fail to process the auditory component compared to the visual component of an audiovisual stimulus (Colavita, 1974), is not evident in 7-year-olds (Nava and Pavani, 2013). Visual and auditory dominance are also task-dependent and in some instances the auditory sense can dominate even in adults (Gori et al., 2012). Other factors that may affect the development of multisensory processing include environmental exposure to and experience of stimuli that commonly “go together” (e.g., Vatakis and Spence, 2007; Thomas et al., 2010), and stimulus salience (e.g., Yuval-Greenberg and Deouell, 2009).

In multisensory research, manipulations of stimulus salience have included the addition of diffuse background noise (e.g., broadband auditory noise or lowered visual contrast). Diffuse background noise of the same modality as targets has been reported to impair processing of unisensory auditory and visual signals (e.g., Kaplan-Neeman et al., 2006; Wagner et al., 2010). Auditory noise has also been linked to impaired visual (Manjarrez et al., 2007), and audiovisual target processing (e.g., Steenken et al., 2008) in both children and adults (Barutchu et al., 2010; Ross et al., 2011; Hillock-Dunn and Wallace, 2012). Such diffuse noise and discrete distractors can affect sensory processing differently. To the best of our knowledge there has been no investigation into the effects of distractors on multisensory processing in children despite its importance in everyday life. Discrete distractors, such as the presence of audiovisual semantically incongruent objects, are common situations of multisensory competition and would be expected to attenuate multisensory processing, particularly for children.

Currently, even in adults, there is little research investigating the effect of unisensory and multisensory distractors on multisensory processing, though the effect of competing incongruent pairs of unisensory auditory and visual stimuli on perceptual processing has been examined (e.g., Colonius and Diederich, 2006). It has also been shown that incongruent stimuli across the sensory systems often interfere with each other in both adults and children (McGurk and Macdonald, 1976). In adults, congruent multisensory speech (Klucharev et al., 2003) and objects (Molholm et al., 2004), and even abstract stimuli with novel associations typically result in multisensory facilitation of RTs and electrophysiological responses (Barutchu et al., 2013), while irrelevant or incongruent audiovisual stimuli (e.g., the sound of a dog and the image of a cat) are often processed more slowly and less accurately than congruent multisensory stimuli (e.g., Klucharev et al., 2003; Molholm et al., 2004; Barutchu et al., 2013). Alsius and Soto-Faraco (2011) have also reported that search times for audiovisual speech are slower when presented with spatially distributed visual distractors compared to auditory distractors that overlapped in both time and space. The authors attributed the results to the different spatial and temporal characteristics inherent in the stimuli of the separate sensory systems (Alsius and Soto-Faraco, 2011). Certainly, multisensory object processing in distracting conditions is likely to rely on basic sensory/perceptual processes that exogenously drive attention, and alert and orient the perceiver (see e.g., Calvert et al., 2004). However, while some research has provided evidence of “near automatic” audiovisual integration in adults (Van Der Burg et al., 2008), others highlight the importance of endogenous attention in supporting multisensory facilitation (e.g., Talsma et al., 2007). Indeed, there is evidence to suggest that attention may influence multiple stages of multisensory processing (see Talsma et al., 2010 for review). The role of “higher order” attention and cognitive resources that underpin skills such as attentional shifting and the suppression of irrelevant information may be particularly important in situations of sensory competition (see Talsma et al., 2010 for review).

“Higher order” attentional and cognitive resources (e.g., Travis, 1998; Konrad et al., 2005), and their cortical architecture are known to undergo protracted maturation periods (e.g., Barnea-Goraly, 2005; Paus, 2005). For example, children show improvement with age (6–10 years) in the ability to suppress distractors on a visual discrimination task (Ridderinkhof et al., 1997). Also, perhaps particularly in children, such higher order resources may be vulnerable to noisy or distracting conditions. For example, auditory working memory was reduced with increasing multitalker babble in children aged 8–10 (Osman and Sullivan, 2014). In summary, given that uni- and multi-sensory responses depend on stimuli and task demands that differ with age, distractors of different sensory modalities are also likely to have different effects on multisensory object recognition while maturing with age.

Knowledge of the effects of multisensory distractors on multisensory object recognition has the potential to inform and promote the development of optimal information processing strategies in complex environments (e.g., school and clinical settings). Thus, the current study aimed to assess the effects of auditory and visual distractors on uni- and multi-sensory object processing in 9- and 11-year-olds, adolescents (aged 15 years) and adults, using an audiovisual discrimination task. We chose to test from 9 years onwards because prior studies have shown that by 9 years of age most children will have multisensory enhancements greater than what can be predicted by race models on simple detection tasks (e.g., Brandwein et al., 2011).

Consistent with the prior research described here, it was predicted that multisensory processing would be immature in children compared to adults and that error rates and RTs would decline with age (e.g., Barutchu et al., 2009; Brandwein et al., 2011) and increase with background auditory distractors, particularly in children (e.g., Barutchu et al., 2010; Ross et al., 2011). For measures of RTs, it was hypothesized that adolescents would perform at similar levels to adults in quiet conditions but not in distracting conditions. Given the reported onset of visual dominance by late childhood (Nava and Pavani, 2013), we expected all groups to show faster visual than auditory RTs in the no distractor condition and that, at least for adults, visual and audiovisual distractors would have a greater impact than auditory distractors on audiovisual object recognition times (Alsius and Soto-Faraco, 2011).

## Materials and methods

### Participants

Seventy-nine children, adolescents and adults participated in the study: 9-year-old children (*n* = 18, 9 females, *M*_age_ = 9:04 years, *SD* = 0:08 years—note that 19 were tested, but one was excluded due to high error rates), 11-year-old children (*n* = 20, 10 females, *M*_age_ = 11:04 years, *SD* = 0:06 years), adolescents (*n* = 19, six females, *M*_age_ = 15:04 years, *SD* = 0:06 years) and adults (*n* = 22, 15 females, *M*_age_ = 26:02 years, *SD* = 2:08 years). Age is denoted in years:months. Children were recruited through a local metropolitan primary school. Male adolescents were recruited through a metropolitan boys' school. Female adolescents and adults were recruited through advertising. Different recruitment of adolescent males and females meant that they were tested in different environments and over a different number of sessions. Children and male adolescents were tested in a quiet schoolroom over 3 or 2 sessions of ~30 or ~45 min, respectively. Female adolescent and adult participants were tested in a single session of ~90 min (with regular breaks) in a quiet laboratory.

There were no differences in accuracy rates or RTs on the audiovisual discrimination task as a function of sex in the adolescent group (*p*-values > 0.08). Thus, adolescent data were pooled across males and females in subsequent analyses. All participants were right handed with no reported history of neurological abnormalities as indicated by adult participants and the parents of children and adolescents. Each adult participant and parent provided informed written consent prior to assessment. Adult and female adolescent participants received a movie voucher for their participation. All procedures were approved by, and complied with, the guidelines of the relevant Human Ethics Committees.

### Sensory and motor screening

To ensure participants were able to see and hear the stimuli used in the audiovisual discrimination task, visual and auditory acuity were assessed. Distance, binocular, and color vision were measured using Patti Pics Mass Vat® Logarithmic Visual Acuity Charts, Randot® Stereotest and Pseudoisochromatic Plates Ishihara Compatible—IPIC®, respectively. An automated test was used to ensure that all participants had the ability to detect pure tones across the frequency range of 250–8000 Hz, in octave steps, at or below 30 dB. Each group was also assessed with the Purdue Pegboard (Tiffin, 1999), to ascertain differences in right hand motor coordination and dexterity. The administration time for screening measures was ~15 min.

### Audiovisual discrimination task

**Figure 1:**
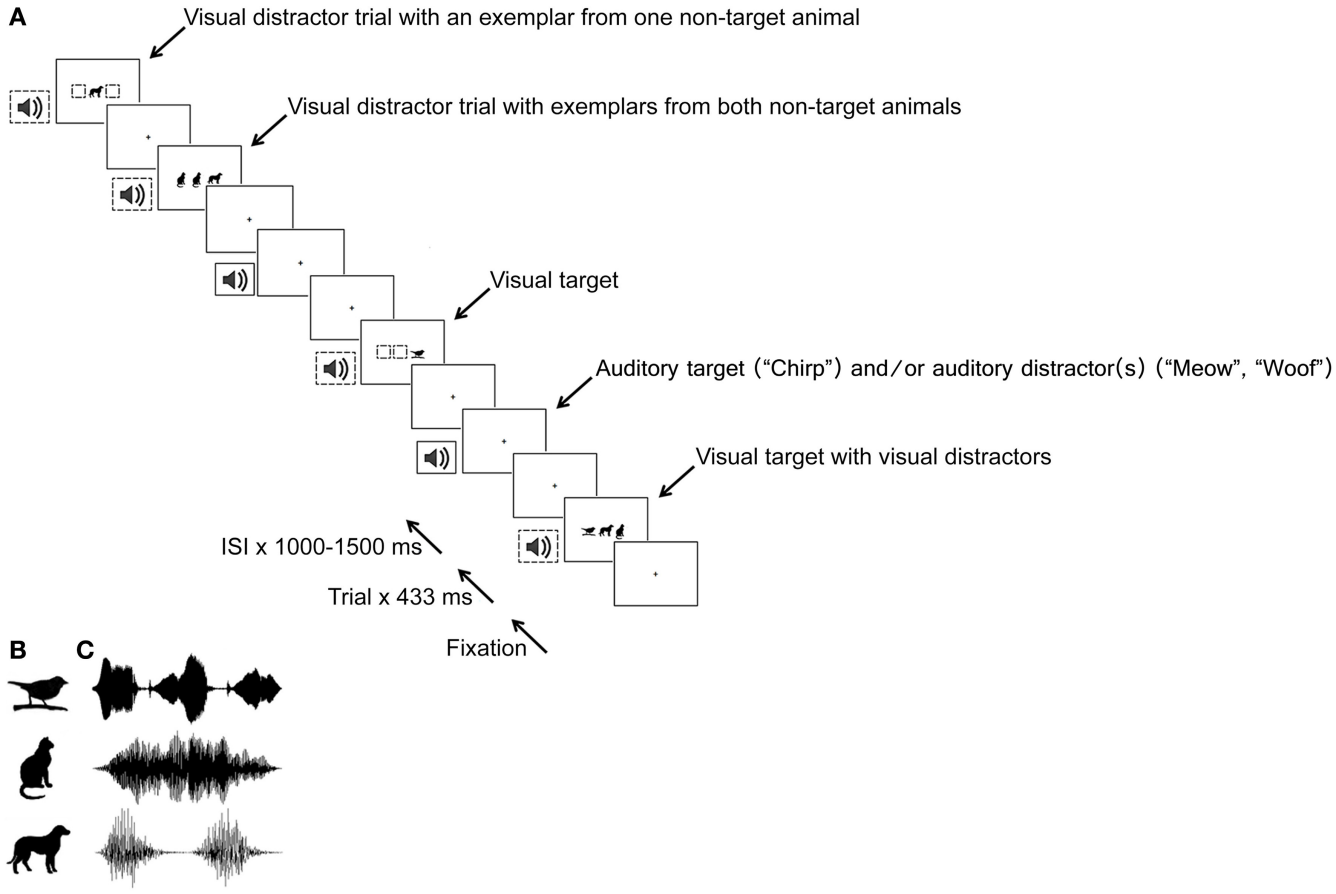
**(A)** A selection of possible trials for blocks in which the bird was the target and the cat and dog were distractors. Empty dashed boxes indicate possible locations of visual stimuli. Auditory targets and distractors could occur with visual targets, visual distractors or in isolation. Visual stimuli are presented in **(B)** and auditory stimuli sound waveforms, which had a duration of 433 ms, are shown in **(C)**, top to bottom: bird, cat, dog. Note: illustrations are not to scale.

An audiovisual discrimination task was used to assess multisensory facilitation. Auditory and visual stimuli comprised objects (i.e., pictures and sounds of a bird, cat, and dog, see Figure 1). Auditory stimuli had different temporal profiles and were easily distinguishable by children and adults when overlapped (see Figure 1 for a representation of the auditory waveforms). The auditory stimuli were delivered using closed-back headphones at 73 dB SPL. Visual stimuli were filled black line drawings (see Figure 1), and had a maximum height and width of 25 mm^2^. They were presented against a white background, using a 17-inch CRT monitor, at one of three positions along the horizontal axis of a central area 76 mm wide (visual angles were ~1.4° for single visual stimuli and ~4.3° along the horizontal axis for multiple visual stimuli). All auditory and visual stimuli were presented for the duration of 433 ms. Onsets of auditory stimuli were synchronized with the 60 Hz refresh rate of the CRT monitor. An oscilloscope was used to ensure that the timing of auditory and visual stimulus onset was consistent (there was less than 1 ms jitter in auditory and visual stimulus onset for multisensory signals).

The auditory and visual stimuli could be either targets or distractors. For any designated target animal (be it bird, cat or dog), the other two animals acted as distractors. Auditory (AT), visual (VT) and audiovisual targets (ATVT) were the presentation of a given animal sound (e.g., bird chirp), picture (e.g., line drawing of a bird) or temporally coincident AT and VT (e.g., sound and picture simultaneously), respectively. The AT, VT, and ATVT were presented without distractors (no distractor, *nd*), or with auditory (*ad*), visual (*vd*), and audiovisual distractors (*advd*) (see Table 1). The *ad* condition comprised animal sounds from the non-target animal stimuli (e.g., when the bird was the target, the cat mew and dog bark acted as auditory distractors); *vd* comprised non-target animal pictures (e.g., the drawing of the cat and dog), and *advd* comprised non-target animal sounds and pictures (note that targets are always denoted with capital letters and distractors with lower case italicized letters). In the attempt to prevent random responding, distractor-alone catch trials were also included and comprised the presentation of non-target uni- and multi-sensory stimuli without targets. Auditory distractor-alone, visual distractor-alone, and audiovisual distractor-alone trials included stimuli from either one (*ad1, vd1, advd1*) or both (*ad2, vd2, advd2*) non-target animals (see Table 1. Note that audiovisual distractors were always congruent pairs, e.g., when the bird was the target, *advd1* comprised either the drawing and sound of the cat or the drawing and sound of the dog, while *advd2* comprised both of these congruent pairs of non-targets). Figure 1 shows an array of possible trials for blocks in which the bird was designated as the target and the other animals as distractors. For each target animal, participants completed a practice block of 20 trials followed by six test blocks of 72 trials, (in total 1296 test trials). Each block of 72 test trials consisted of the random presentation of 36 target trials (three of each of the 12 target × distractor stimulus combinations) and 36 distractor-alone trials (i.e., 50% target ratio). The inter-stimulus period was also randomly varied between 1500 and 2000 ms in 50 ms intervals.

**Table 1:** **Target stimulus × distractor and distractor-alone catch trial types**.

**Distractor conditions**	Target trials^*^			**No. distractor-alone trials**	
	**AT**	**VT**	**ATVT**	**1**	**2**
*nd*	AT*nd*	VT*nd*	ATVT*nd*	*–*	*–*
*ad*	AT*ad*	VT*ad*	ATVT*ad*	*ad1*	*ad2*
*vd*	AT*vd*	VT*vd*	ATVT*vd*	*vd1*	*vd2*
*advd*	AT*advd*	VT*advd*	ATVT*advd*	*advd1*	*advd2*

* *Targets: AT, auditory target, VT, visual target; ATVT, audiovisual target. Distractors: nd, no distractor; ad, auditory distractor; vd, visual distractor and advd, audiovisual distractor. Trials with visual stimuli only ever comprised one or three visual images (in order to eliminate visual eccentricity as a cue for invalid advd2 trials. See Figure 1 for an example). Trials with auditory stimuli comprised one, two or three stimuli (temporally coincident). Distractor-alone trials comprised stimuli from one (1) or two (2) non-target animals (e.g., when the bird was the target, ad1 refers to the presentation of the sound of the cat or the dog, while ad2 refers to the sound of the cat and the dog; similarly, vd1 refers to the picture of the cat or the dog and vd2 refers to the picture of the cat and the dog with one of them repeated (to ensure three stimuli were presented in both target-present (ATadvd, VTadvd, ATVTadvd) and target absent advd2 trials)*.

### Procedure

All participants had their vision and hearing assessed first, followed by assessment of motor coordination and dexterity. Participants sat ~1 m from the CRT screen in the audiovisual discrimination task and were asked to fixate on a centrally appearing cross (the visual angle of the fixation cross was ~0.5°). Participants were instructed to make a button press as quickly and accurately as possible with their right index finger when they heard or saw the target animal (i.e., when the target occurred anywhere in the display), and to make no response when the target animal was absent. The designated order of targets (i.e., bird, cat, and dog) was counterbalanced across participants. Each individual completed the full set of six blocks for a given animal before those for the next animal (male adolescents completed blocks for one animal in the first session and the remaining two animals in the second session, while children completed blocks for a different animal in each session). Breaks were offered between each block, or as required. Motor response accuracy and speed were recorded.

## Results

### Screening measures

All participants reached criteria on screening measures for normal audition and vision. However, (as noted above) one participant in the 9-year-old group was excluded, due to error rates above 70%, in some conditions on the audiovisual discrimination task.

To assess differences in motor coordination and dexterity between groups on the Purdue Pegboard, we used a One-Way Analysis of Variance (ANOVA). Motor coordination and dexterity improved with age, *F*_(3, 75)_ = 18.48, *p* < 0.001, η^2^ = 0.43. There were no significant differences between 9-year-olds (*M* = 12.98, *SD* = 1.73) and 11-year-olds (*M* = 14.45, *SD* = 1.33), or 11-year-olds and adolescents (*M* = 15.07, *SD* = 1.80), who performed significantly better than 9-year-olds. Adults performed significantly better than both children and adolescents (*M* = 17.20, *SD* = 2.27).

### Analysis of accuracy and RTs for the audiovisual discrimination task

For the audiovisual discrimination task, RTs less than 100 ms and greater than 1499 ms (i.e., greater than the lower bound of the ISI) were excluded from all subsequent analyses. Based on these criteria, 4.16, 2.27, 0.76, and 0.13% of target trials were excluded for 9-year-olds, 11-year-olds, adolescents and adults, respectively.

For all ANOVAs, where appropriate, a Greenhouse-Geisser or Huynh-Feldt correction was used to adjust degrees of freedom for violations of the assumption of sphericity. Significant interaction effects were followed by “simple effects” analyses using pair-wise comparisons (e.g., Howell and Lacroix, 2012). Given the large number of comparisons with a 4 (Group) × 3 (Target Stimulus) × 4 (Distractor) design, a Bonferroni adjustment was considered too conservative. Therefore, the alpha level was adjusted to 0.01 for all analyses (Keppel and Zedeck, 1989). Individual *p*-values are reported for main and interaction effects but not for follow-up *post-hoc* simple effects analyses, for which all reported significant results correspond to *p*-values < 0.01.

### Accuracy rates

The audiovisual discrimination task used here was developed to assess developmental trends in RTs. However, as error rates in audiovisual detection have previously been shown to be higher in children than adults, error rates on the audiovisual discrimination task were also assessed first.

Overall, percentage error rates were low for all groups, with strong violations of normality in many conditions. Thus, non-parametric statistics were used to assess false alarms (i.e., errors of commission for invalid (non-target) trials) and misses (i.e., errors of omission to target stimuli). Group differences were assessed using Kruskal–Wallis tests, with *post-hoc* comparisons using Mann–Whitney *U*-tests. Within group differences were assessed using Friedman tests, with follow-up comparisons using Wilcoxon Signed Ranks tests. An alpha level criterion of 0.01 was applied throughout.

**Figure 2:**
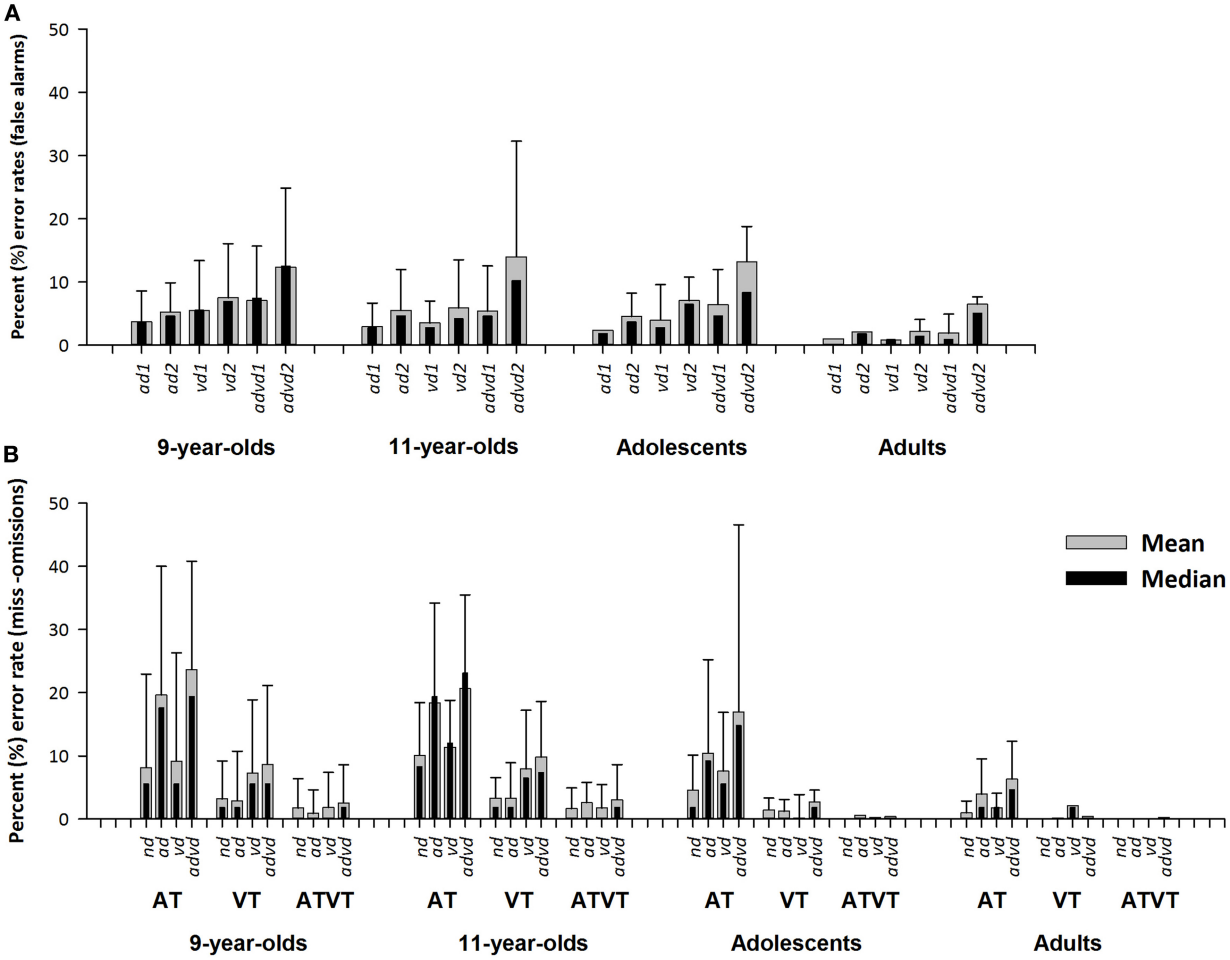
**(A)** Mean and median percentage (±interquartile range) errors rate (false alarms) to auditory, visual and audiovisual distractor-alone trials with stimuli from one (auditory, *ad1*, visual, *vd1*, audiovisual *advd1*) or both (auditory, *ad2*, visual, *vd2*, audiovisual *advd2*) non-target animals, for the groups, 9-year-olds, 11-year-olds, adolescents, and adults. **(B)** Mean and median percentage (±interquartile range) error rate (misses) for auditory (AT), visual (VT) and audiovisual targets (ATVT) in the no distractor (*nd*), and auditory (*ad*), visual (*vd*), and audiovisual distractor (*advd*) conditions for the groups, 9-year-olds, 11-year-olds, adolescents, and adults.

As shown in Figure 2A, false alarm error rates were higher for children and adolescents than adults. There were significant group differences for the *ad1*, χ^2^_(3)_ = 12.52, *p* = 0.006; *ad2*, χ^2^_(3)_ = 12.02, *p* = 0.007; *vd1*, χ^2^_(3)_ = 20.78, *p* < 0.001; *vd2*, χ^2^_(3)_ = 20.95, *p* < 0.001; and *advd1*, χ^2^_(3)_ = 16.21, *p* = 0.001 conditions. Group differences failed to reach the alpha 0.01 criterion for *advd2*, χ^2^_(3)_ = 9.78, *p* = 0.021. False alarm error rates were significantly higher in children than adults. Adolescents performed at adult levels only in *ad* conditions.

Misses also decreased with age (see Figure 2B). Significant differences were observed between the four groups for all target stimulus and distractor conditions: AT*nd*, χ^2^_(3)_ = 35.98, *p* < 0.001; VT*nd*, χ^2^_(3)_ = 28.24, *p* < 0.001; ATVT*nd*, χ^2^_(3)_ = 16.55, *p* < 0.001; AT*ad*, χ^2^_(3)_ = 26.04, *p* < 0.001; VT*ad*, χ^2^_(3)_ = 22.71, *p* < 0.001; ATVT*ad*, χ^2^_(3)_ = 12.19, *p* = 0.007; AT*vd*, χ^2^_(3)_ = 31.94, *p* < 0.001; VT*vd*, χ^2^_(3)_ = 36.56, *p* < 0.001; ATVT*vd*, χ^2^_(3)_ = 15.4, *p* = 0.002; AT*advd*, χ^2^_(3)_ = 24.18, *p* < 0.001; VT*advd*, χ^2^_(3)_ = 45.97, *p* < 0.001; ATVT*advd*, χ^2^_(3)_ = 25.99, *p* < 0.001. To summarize, 9- and 11-year-old children performed at similar levels of miss-rates to each other, and on the whole performed worse than adults. Adolescents performed at adult levels for ATVT but remained immature for AT (in *nd* and *vd*) and VT (in all distractor conditions).

For all groups, miss rates to auditory, visual and audiovisual targets were modulated by distractor modality. All groups recorded a main effect of distractor condition on miss rates to AT: 9-year-olds, [χ^2^_(3)_ = 30.82, *p* < 0.001]; 11-year-olds, [χ^2^_(3)_ = 16.79, *p* = 0.001]; adolescents [χ^2^_(3)_ = 30.25, *p* < 0.001]; and adults [χ^2^_(3)_ = 27.95, *p* < 0.001]. For 9- and 11-year-olds, AT miss rates were higher in the auditory and audiovisual distractor conditions than the no distractor and visual distractor conditions. For adolescents and adults, AT error rates were higher in the audiovisual distractor condition compared to other distractor conditions, and also higher in the auditory distractor condition compared to the no distractor condition.

There was a main effect of distractor condition on miss rates to VT for 9- and 11-year-olds [χ^2^_(3)_ = 21.09, *p* < 0.001; and χ^2^_(3)_ = 32.49, *p* < 0.001, respectively] but not for adolescents [χ^2^_(3)_ = 9.52, *p* = 0.023, using the alpha 0.01 criterion], or adults [χ^2^_(3)_ = 3.00, *p* = 0.392]. For children, VT miss rates were higher in the visual and audiovisual distractor conditions compared with the no distractor and auditory distractor conditions.

Only 9-year-old children showed a significant main effect of distractor condition on miss rates to ATVT: 9-year-olds, [χ^2^_(3)_ = 12.58, *p* = 0.006]; 11-year-olds, [χ^2^_(3)_ = 7.20, *p* = 0.066]; adolescents, [χ^2^_(3)_ = 2.31, *p* = 0.510]; and adults, [χ^2^_(3)_ = 9.00, *p* = 0.029, using the alpha 0.01 criterion]. However, none of the comparisons reached significance using the 0.01 alpha level for the 9-year-old group (a trend for significantly higher error rates was noted for the audiovisual compared with the auditory distractor condition, *p* = 0.014). In sum, all groups showed similar patterns of omission rates across distracting conditions. The greatest impact of distractors tended to be on unisensory targets, which were specifically impacted by distractors of the same modality and by audiovisual distractors. No group showed significant differences in omission rates to audiovisual targets across distractor conditions.

### Reaction times

Only correct responses were included in analyses of RTs. To assess differences between unisensory and audiovisual targets across distractor conditions, mean RTs were analyzed using a Three-Way mixed Group (9-year-olds, 11-year-olds, adolescents, and adults) × Target Stimulus (AT, VT, and ATVT) × Distractor (*nd*, *ad*, *vd*, and *advd*) ANOVA (Howell and Lacroix, 2012).

**Figure 3:**
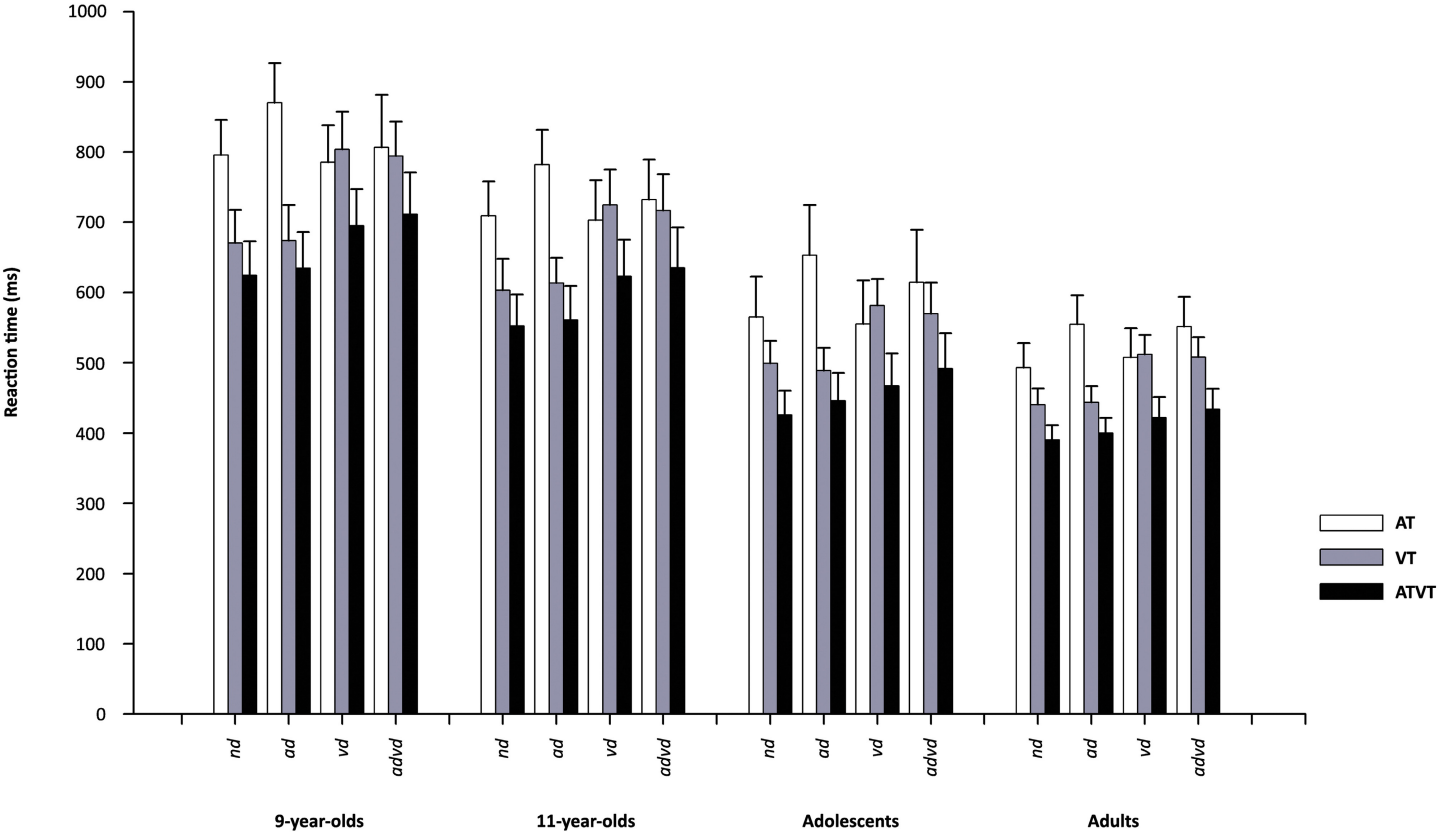
Mean reaction times (RTs; ±95%CI) for auditory (AT), visual (VT) and audiovisual (ATVT) targets in the no distractor (*nd*), auditory distractor (*ad*), visual distractor (*vd*) and audiovisual distractor (*advd*) conditions for the groups, 9-year-olds, 11-year-olds, adolescents and adults.

As can be seen in Figure 3, there was a similar pattern of RT interaction between target type and distractor modality for all age groups. However, RTs differed between groups, with 9- and 11-year-olds being slower than adolescents, who performed close to mature (adult) levels. The mixed ANOVA showed significant main effects of target, *F*_(1.27, 95.52)_ = 434.84, *p* < 0.001, η^2^ = 0.85, distractor, *F*_(2.6, 195.03)_ = 161.78, *p* < 0.001, η^2^ = 0.68, and group, *F*_(3, 75)_ = 35.06, *p* < 0.001, η^2^ = 0.58. There was a significant two-way interaction between target × distractor, *F*_(3.83, 287.13)_ = 145.34, *p* < 0.001, η^2^ = 0.66. However, the interaction between group × distractor only approached significance according to our criterion of 0.01, *F*_(7.80, 195.03)_ = 2.35, *p* = 0.021, η^2^ = 0.09, and the interaction between group × target was not significant, *F*_(3.821, 95.52)_ = 1.47, *p* = 0.218. The omnibus three-way interaction of group × target × distractor was significant, *F*_(11.49, 287.13)_ = 5.4, *p* < 0.001, η^2^ = 0.18.

All groups recorded audiovisual facilitation in all distractor conditions, with significantly faster RTs for ATVT than AT and VT. For unisensory targets, RTs for VT were significantly faster than AT in the no distractor and auditory distractor condition but did not differ significantly in the visual distractor and audiovisual distractor conditions for all groups. For the distractor conditions, AT RTs were significantly slower in the auditory distractor than all other distractor conditions for children and adolescents. Adolescents' RTs for AT were also significantly slower in the audiovisual distractor condition compared to the no distractor and visual distractor conditions. For adults, RTs for AT were significantly slower in the auditory distractor and audiovisual distractor conditions compared to the no distractor and visual distractor conditions (but did not differ significantly between the audiovisual distractor and auditory distractor conditions). All groups recorded significantly slower RTs for VT in the visual distractor and audiovisual distractor conditions compared to the no distractor and auditory distractor conditions. For 9-year-olds, 11-year-olds and adults, ATVT RTs were significantly slower in the visual distractor and audiovisual distractor conditions compared to the no distractor and auditory distractor conditions. For adolescents, RTs for ATVT differed significantly in all conditions, being fastest to slowest in the no distractor, auditory distractor, visual distractor and audiovisual distractor conditions.

Nine-year-olds were significantly slower than 11-year-olds for VT RTs in the no distractor and visual distractor conditions, and ATVT RTs in the no distractor and auditory distractor conditions. There were no other significant differences between children's RTs. Children recorded significantly slower RTs than adolescents and adults in all Target × Distractor conditions. Adolescents' RTs were significantly slower than adults' only for AT in the auditory distractor condition (*p* < 0.01 for all comparisons).

In summary, all groups showed (1) multisensory facilitation in all distractor conditions (i.e., faster RTs to ATVT than AT or VT, even in the presence of auditory and/or visual distractors) (2) visual dominance in the no distractor condition, and (3) similar impacts of distractors on RTs, including that compared to the no distractor condition, RTs to AT were slower in the auditory distractor condition and RTs to VT and ATVT were slower in the visual and audiovisual conditions. Adolescents and adults had faster RTs than children.

Sensory dominance, defined here by faster average RTs to visual or auditory targets, was calculated for each individual in each distractor condition. Group differences in the proportion of participants demonstrating visual or auditory dominance were assessed using Kruskal–Wallis tests. As there were no significant differences between the groups, these data were collapsed across age for analysis of differences between distractor conditions, using Friedman tests, with follow-up comparisons by Wilcoxon Signed Ranks tests.

**Figure 4:**
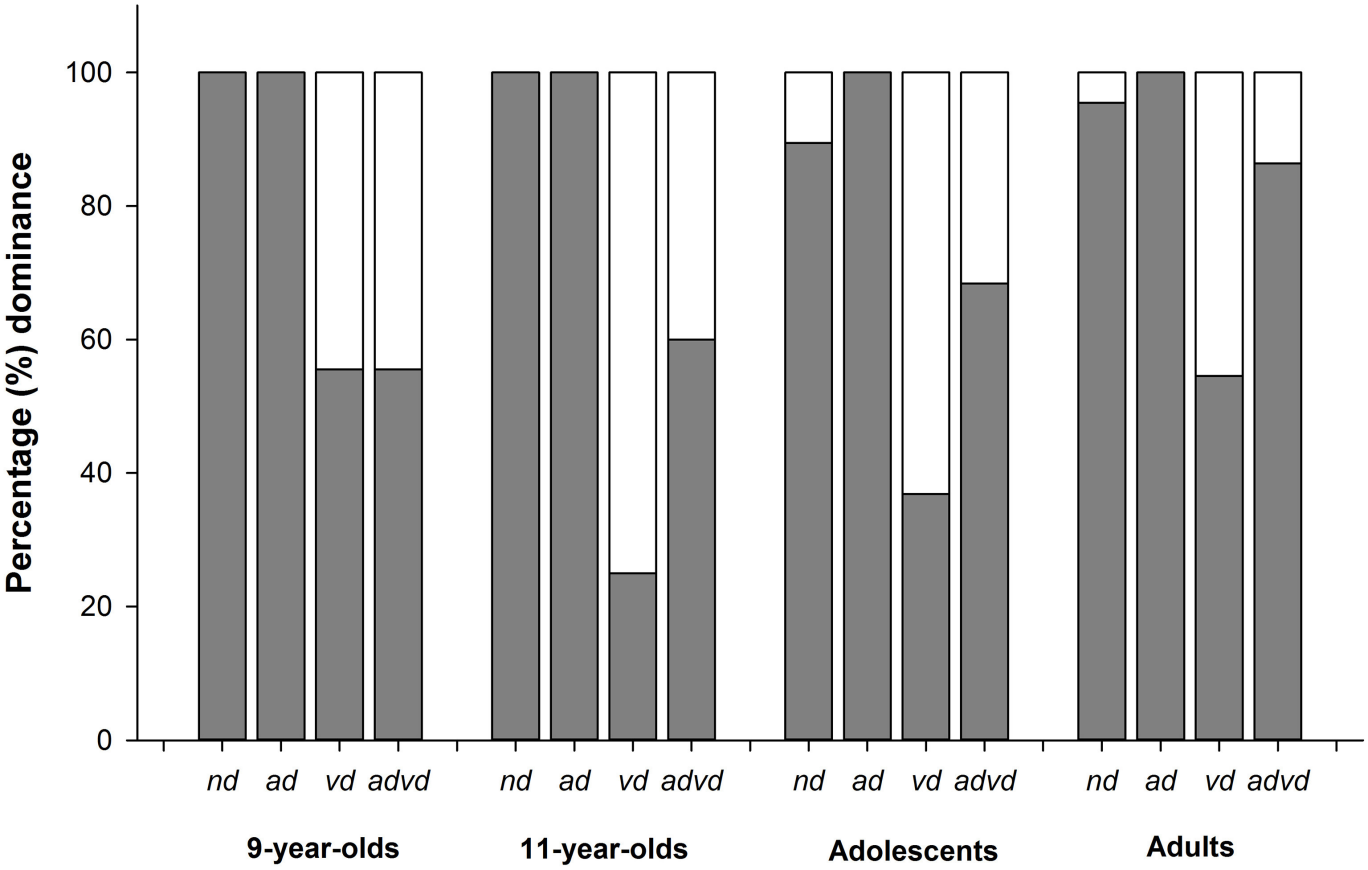
The percentage of participants in each group showing visual and auditory dominance (i.e., faster mean RTs to visual or auditory targets) for the four distractor conditions: no distractor (*nd*), auditory distractor (*ad*), visual distractor (*vd*) and audiovisual distractor (*advd*) conditions. Gray portion represents visual dominance. White portion represents auditory dominance.

Figure 4 demonstrates that all groups recorded a high percentage of participants showing visual dominance (i.e., faster mean RTs for visual target stimuli than for auditory target stimuli). There were no significant group differences in the proportion of visual dominance for each distractor condition: *nd* [χ^2^_(3)_ = 3.84, *p* = 0.279], *ad* [χ^2^_(3)_ = 0.000, *p* = 1.0], *vd* [χ^2^_(3)_ = 4.854, *p* = 0.183], and *advd* [χ^2^_(3)_ = 5.24, *p* = 0.155]. Also, for all groups, visual dominance was reduced in the visual distractor condition (even more than in the audiovisual distractor condition). When collapsed across age groups, rates of visual dominance differed significantly between all distractor conditions (at *p* < 0.001) except *nd* and *ad* (*p* = 0.083), being highest to lowest in the order of *nd* and *ad*, *advd, vd*.

### Test of the race model and variability

**Figure 5:**
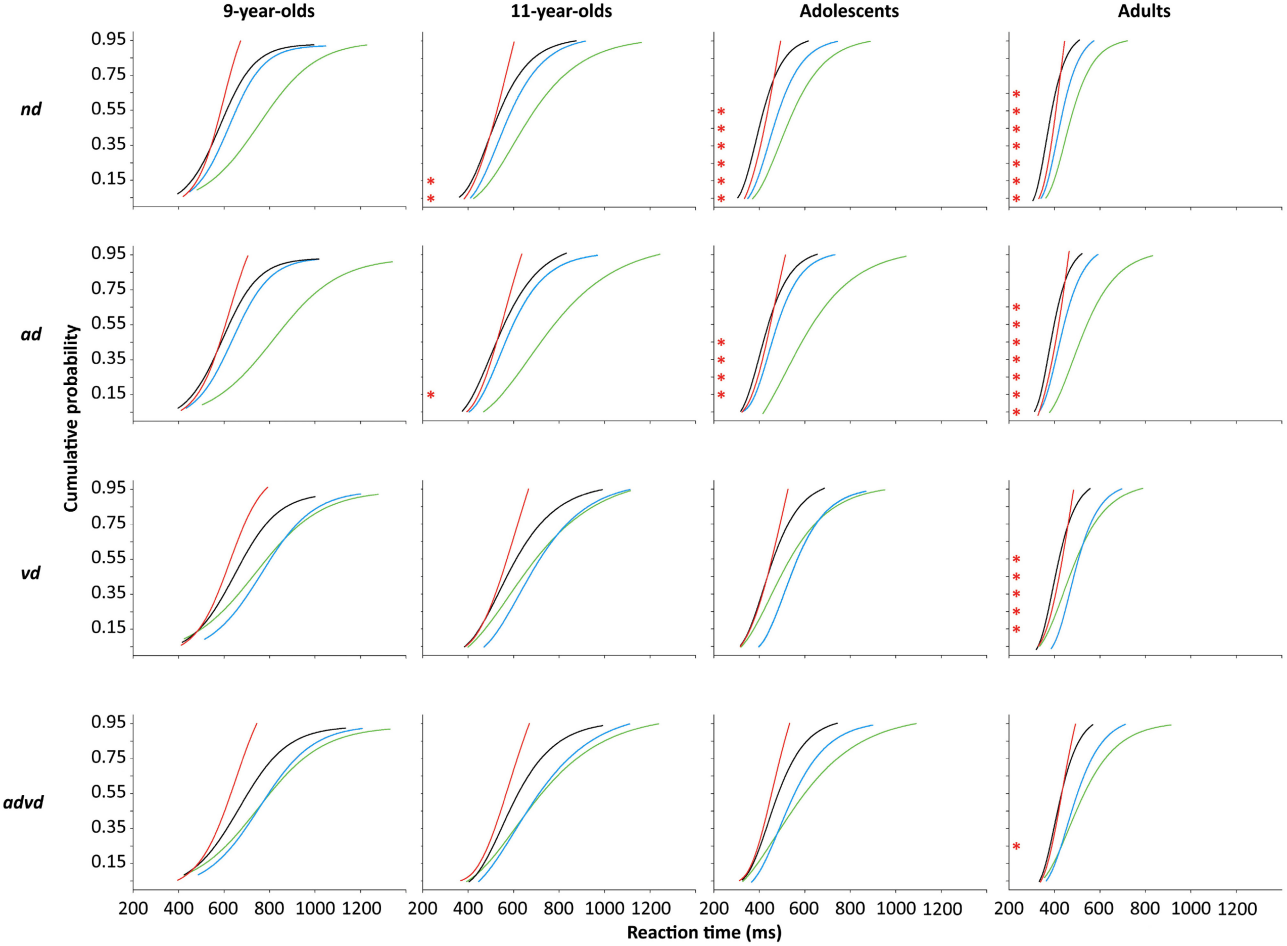
Mean cumulative density functions (CDFs) of reaction times (RTs) to auditory (AT, green line), visual (VT, blue line), and audiovisual (ATVT, black line) targets in the no distractor (*nd*), and auditory (*ad*), visual (*vd*) and audiovisual (*advd*) distractor conditions, for the groups 9-year-olds, 11-year-olds, adolescents, and adults. Red line represents the CDF of the bound (AT+VT). Asterix (^*^) indicates a significant violation of the race model predicted inequality (Miller, 1982).

Race models are traditionally tested by assessing violations of Miller's race model inequality that limits the distribution of audiovisual RTs to the probability of the sum of unisensory cumulative response distribution functions (Miller, 1982). When the inequality is violated (i.e., multisensory responses exceed probability summation) the traditional inference has been that resources have been “pooled” or “integrated” across the sensory systems (Miller, 1982; Ulrich et al., 2007). As many multisensory interactions have been shown to occur within the bounds of probability summation (i.e., at the level of “sub-additivity” or “additivity”; for review see Stanford and Stein, 2007), the test of the race model inequality is understood to be a conservative estimate of the magnitude of multisensory facilitation (Townsend and Honey, 2007). On the other hand, recent studies have suggested that violations of race models could be explained by increased variance of motor responses under multisensory conditions (Otto and Mamassian, 2012), thus, violating the “context invariance assumption” of Miller's inequality test (i.e., that processing of a stimulus in one modality will be unaffected by the concurrent presentation of stimuli in another modality) (Ashby and Townsend, 1986; Townsend and Wenger, 2004). To determine whether multisensory facilitation violated probability summation, as defined by violations of the race model inequality (Miller, 1982), cumulative density functions (CDFs) were created for audiovisual and sum of unisensory RTs (see Ulrich et al., 2007 for complete procedural details). The CDFs depicted in the ogive figures presented in Figure 5 were fitted to the probabilities of 0.05, 0.15, 0.25, 0.35, 0.45, 0.55, 0.65, 0.75, 0.85 and 0.95. For each distractor condition, the Three-Way interaction effect of a mixed model ANOVA with the within subjects factors Target Stimulus (ATVT and sum of AT+VT) and Probability (0.05 to 0.95), and the between subjects factor Group (9-year-olds, 11-year-olds, adolescents, adults) was assessed. *Post-hoc* analyses were carried out to compare ATVT and sum of AT+VT CDFs at each probability.

As demonstrated in Figure 5, violations of the race model inequality were modulated as a function of age and distractor condition. For each distractor condition, the interaction effects of the Three-Way ANOVAs comparing Group × Stimulus × Probability were significant (see Table 2). *Post-hoc* analyses indicated that race models could account for 9-year-olds' responses in all distractor conditions; although the ATVT CDF appeared faster than the AT+VT CDFs in the no distractor and auditory distractor conditions (see Figure 5), these differences were not statistically significant. In the no distractor condition, 11-year-olds violated the race model for probabilities of 0.05 to 0.15. As can be seen in Figure 5, responses in excess of probability summation were at near-adult levels for adolescents in the no distractor condition, with adolescents recording violations for probabilities of 0.05 to 0.55, and adults from 0.05 to 0.65. By contrast, in the auditory distractor condition, 11-year-olds and adolescents both showed fewer violations of probability summation than adults: 11-year-olds recorded violations for the 0.15 probability, and adolescents recorded violations from 0.15 to 0.45, while adults recorded violations from 0.05 to 0.65. Only adults recorded violations in the visual distractor and the audiovisual distractor conditions (from 0.15 to 0.55 and for the 0.25 probability, respectively), in which children's and adolescents' responses could be accounted for by race models.

**Table 2:** **F-statistic (Greenhouse-Geisser corrected degrees of freedom), *p*-values (p), and effect size (η^2^) for interaction effects of Three-Way mixed model ANOVAs comparing ATVT and AT+VT mean CDFs at 10 probabilities (0.05 to 0.95 in 0.05 increments) for the four groups (9-year-olds, 11-year-olds, adolescents, and adults) for the four distractor conditions: no distractor (nd); auditory distractor (ad), visual distractor (vd) and audiovisual distractor (advd)**.

**Distractor condition**	***DF***	***F***	***p***	**η^2^**
*nd*	5.21, 130.33	15.65	<0.001	0.39
*ad*	4.99, 124.86	13.84	<0.001	0.36
*vd*	6.46, 161.40	19.63	<0.001	0.44
*advd*	6.44, 161.10	17.52	<0.001	0.41

As can be observed in the ogive graphs (i.e., the CDFs) presented in Figure 5, an increase in variability of RTs cannot explain all the observed violations in race models, nor the lack of violations in some cases. An analysis of RT variance using the Coefficient of Variation (Cv) further confirmed that variance was more likely to be lower for multisensory than unisensory target RTs. The coefficient of variation of each participant's RTs was calculated for each target by distractor condition separately. Group means were analyzed using a Three-Way mixed model ANOVA (group × stimulus × distractor). The ANOVA was followed by planned comparisons to assess whether variability for the ATVT stimulus was significantly greater than the unisensory AT and VT stimuli as predicted by Otto and Mamassian (2012).

**Figure 6:**
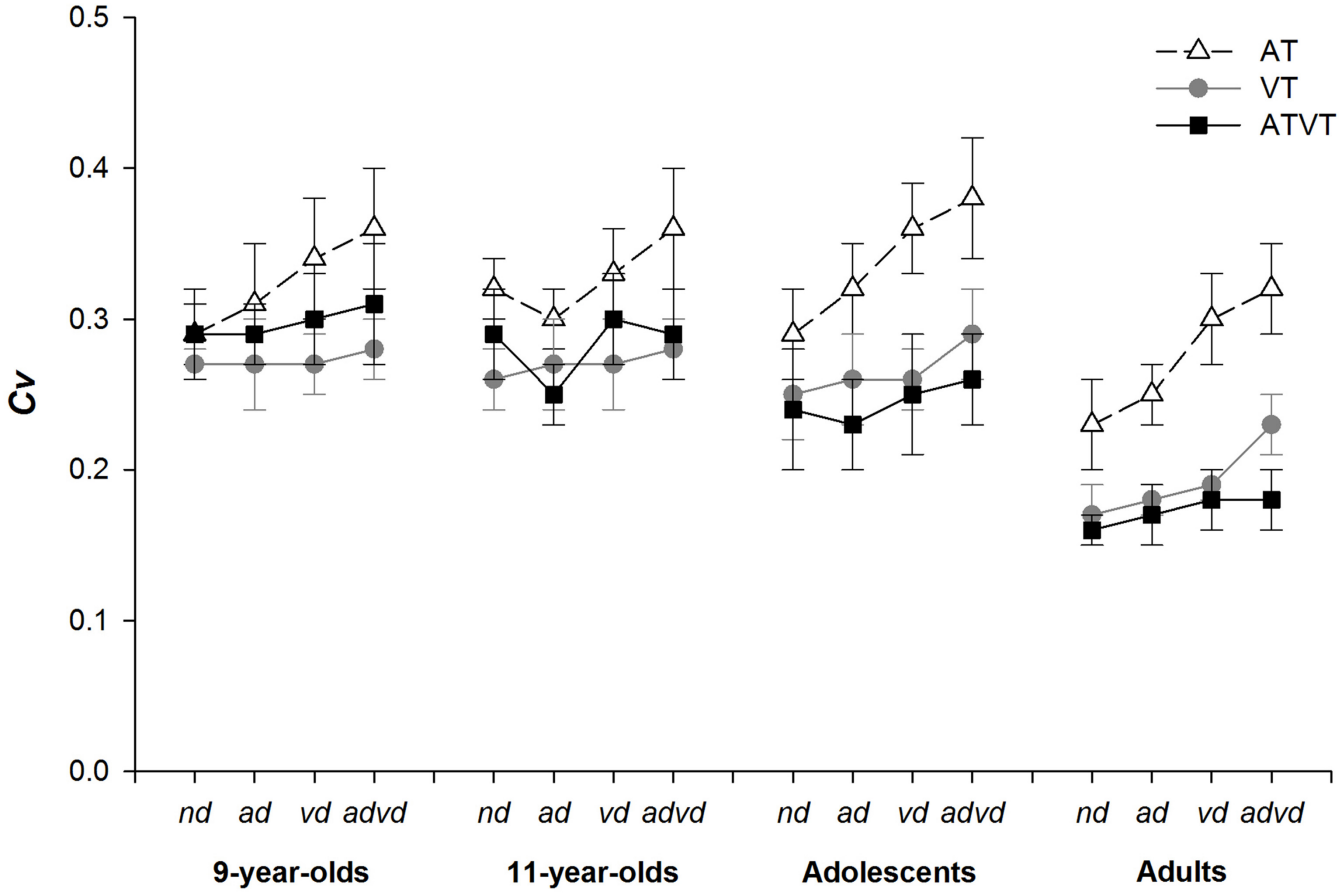
Coefficient of variation (*Cv*, ± 95% *CI*) for auditory (AT), visual (VT) and audiovisual (ATVT) targets in the no distractor (*nd*), auditory distractor (*ad*), visual distractor (*vd*) and audiovisual distractor (*advd*) conditions for the groups, 9-year-olds, 11-year-olds, adolescents, and adults.

As shown in Figure 6, variability was never as high for audiovisual RTs as for auditory RTs and variability tended to decrease with age. The Three-Way ANOVA revealed significant main effects of group, *F*_(3, 75)_ = 19.99, *p* < 0.001, η^2^ = 0.44, stimulus, *F*_(2, 150)_ = 152.90, *p* < 0.001, η^2^ = 0.67, and distractor conditions, *F*_(3, 225)_ = 44.78, *p* < 0.001, η^2^ = 0.37. There were significant two-way interactions between group × stimulus, *F*_(6, 150)_ = 9.25, *p* < 0.001, η^2^ = 0.27, and stimulus × distractor, *F*_(6, 450)_ = 9.03, *p* < 0.001, η^2^ = 0.11. There were no significant interactions between group × distractor, *F*_(9, 225)_ = 1.91, *p* = 0.051, or group × stimulus × distractor, *F*_(18, 450)_ = 1.22, *p* = 0.238. In contrast to the predictions of Otto and Mamassian (2012), planned comparisons between ATVT and the unisensory AT and VT stimuli showed that for all groups, variance measures for ATVT stimuli were never significantly greater than unisensory AT or VT stimuli. This pattern held for all distractor conditions.

## Discussion

The present study provides new evidence for the robust nature of multisensory facilitation to meaningful objects, even in the presence of competing auditory and visual distracting objects. Consistent with our hypotheses, adolescents recorded near-adult levels of multisensory facilitation in the no distractor condition, and children and adolescents, compared to adults, showed a restricted range of violations of the race model with auditory distractors (with no evidence of violations of the race model inequality for the youngest, 9-year-old, group). There were also no violations of the race model with visual and audiovisual distractors for children or adolescents. Distractors, specifically visual and audiovisual distractors, negatively impacted RTs, and otherwise robust visual dominance patterns in all groups, including adults.

As indicated above, our results, like most recent reports in the literature, demonstrated developmental trends of faster RTs to simple auditory and visual stimuli, and in turn, improvements in multisensory processing with age from childhood to adulthood (e.g., McGurk and Macdonald, 1976; Gori et al., 2008; Nardini et al., 2008, 2013; Barutchu et al., 2009; Hillock-Dunn and Wallace, 2012; Nava and Pavani, 2013; Petrini et al., 2014) in both no added noise (e.g., Brandwein et al., 2011) and noisy conditions (e.g., Barutchu et al., 2010; Ross et al., 2011). The current study also demonstrated that adolescents and particularly children had higher error rates than adults (see Figure 2), suggesting that they were likely to have found the discrimination task more difficult. On screening, there were also the expected improvements in motor coordination and dexterity with age, with better dexterity being seen in adolescents than in 9-year-olds. Nonetheless, 11-year-old children and adolescents performed similarly on the motor coordination and dexterity measure, yet adolescents' motor responses were faster on the audiovisual discrimination task. Therefore, an immaturity in the motor system is unlikely to account for multisensory developmental effects *per-se*.

### Effect of distractors on RTs

Despite developmental improvements in RTs with age, multisensory processing of objects appears to be constrained by consistent cross-sensory rules from at least 9 years of age (see e.g., Stein et al., 1988). For example, RTs to unisensory targets were slower with same-modality but not with cross-modal distractors when compared to the no distractor condition for all age groups. Such findings are consistent with the idea that competition within sensory modalities has a greater effect on processing than competition across sensory modalities (e.g., Talsma et al., 2006). The results are also consistent with adult literature demonstrating no significant increase in RTs for incongruent audiovisual objects compared with RTs for unisensory objects (e.g., Molholm et al., 2004; however, see e.g., Laurienti et al., 2004). Taken together, these findings support the idea of some early separation between attentional processing systems for audition and vision (Alais et al., 2006). On the other hand, the multisensory facilitation of RTs seen here in all distractor conditions points to interactions between sensory systems.

In the present study, all groups demonstrated visual dominance, with faster motor responses to visual than auditory objects in the no distractor condition, consistent with reports of a gradual developmental shift in sensory processing and object recognition to vision throughout childhood (e.g., Nava and Pavani, 2013). Thus visual dominance may explain the relatively greater impact of visual and audiovisual distractors on audiovisual RTs. This explanation is consistent with Alsius and Soto-Faraco (2011) who reported audiovisual speech processing to be impaired with increasing number of visual but not auditory distractors in adults. These effects are presumably related not only to differences in salience of visual compared to auditory distractors, but also to the fact that visual processing requires shifts in spatial attention, whereas the auditory distractors, such as speech and the object related sounds used here, can be processed for content rather than location (Alsius and Soto-Faraco, 2011; however, see Spence et al., 2000). It is important to note that in the present study while differences in spatial and temporal properties of the auditory and visual stimuli may provide an alternative explanation for the greater effects of visual than auditory distractors on audiovisual RTs, error rates were highest for conditions with auditory distractors (see Figure 2), suggesting auditory distractors were as, if not more, distracting than visual distractors, yet had much less of an impact on multisensory target processing. Indeed visual dominance for familiar object recognition was expected here given the duration of presentation time needed for recognition of meaningful sounds (e.g., Koppen et al., 2008; compared to faster RTs to simple auditory sounds such as beeps reported by Miller, 1982). The observed findings may also be partly related to the familiarity of the objects used as target and distractor stimuli, and may be driven by higher order (top-down) processes (e.g., Barutchu et al., 2013). Further research needs to investigate whether similar patterns of sensory interference and dominance will be observed with novel targets and distractors.

Importantly, the present study expands on developmental research by demonstrating that visual dominance of attention and action is vulnerable particularly in the presence of visual or audiovisual distractors. This finding aligns with research that postulates flexibility of sensory dominance under degraded conditions in adults (e.g., Alais and Burr, 2004; Yuval-Greenberg and Deouell, 2009), suggesting that visual dominance is partly dependent on environmental conditions. Future research would benefit from manipulating the spatial and temporal characteristics of both auditory and visual stimulus components on unisensory and multisensory object recognition. The specific relationship between attention, distractors and multisensory facilitation in development also requires future physiological investigation. Certainly all age groups demonstrated similar trends in the interactions between the sensory modalities of targets and distractors (for accuracy rates as well as for RTs) suggesting several possibilities from the prior literature. For example Van der Burg and colleagues suggest that aspects of multisensory facilitation may be near-automatic, i.e., driven by involuntary attention (Van Der Burg et al., 2008). On the other hand, further application of selective attention may be required under conditions of sensory competition or increased perceptual load (Alsius et al., 2005; also see Lenartowicz et al., 2014). There may also be rapid spreading of attention between sensory modalities, presumably on the basis of higher order factors such as amodal object representations (e.g., Fiebelkorn et al., 2010). Overall, the nature of these observed similarities across development supports the reported complexity in interactions between all the senses.

### Multisensory facilitation and violations of the race model assumptions

The observation that all groups showed multisensory facilitation of mean RTs in all distractor conditions (see Figure 3), demonstrates the early onset of clear behavioral benefits of audiovisual interactions. However, facilitation did not correspond to violations of the race model in the visual distractor and audiovisual distractor conditions for 11-year-old children and adolescents, and in any distractor condition for 9-year-old children (see Figure 5). The currently observed fewer violations of the race model inequality in children and adolescents than in adults is consistent with research demonstrating variable or suboptimal multisensory interactions throughout childhood and early adolescence (Gori et al., 2008; Nardini et al., 2008, 2013; Barutchu et al., 2009), as well as being broadly consistent with the onset of adult levels of multisensory interactions (in non-distracting conditions) around 14 years of age (Brandwein et al., 2011). Thus our results add to the body of literature that has shown developmental trends with age in multisensory interactions in complex environments from background auditory noise (Barutchu et al., 2010; Ross et al., 2011; Hillock-Dunn and Wallace, 2012). Of particular note, our results extend the observation of developmental effects in multisensory processing to the recognition of common meaningful objects with auditory, visual and audiovisual distractors. It is important to note that the immaturities currently seen in children and adolescents may be related to multisensory processing and its dependence on factors such as attentional control. The developmental pattern in violations of the race model, and the very limited violations in the audiovisual distractor condition by adults, is likely to be consistent with research that highlights the impact of perceptual loads and increasing task difficulty on attentional and multisensory processing (see Lavie, 2005; Benoni and Tsal, 2012). Indeed, previously it has been argued that the amount of information available at onset of a response is likely to be a key factor in whether RTs exceed probability summation measured by violations of the race model (Rowland and Stein, 2007; Rowland et al., 2007). Perhaps in our case, the amount of information available at response onset might be compromised, not only by stimulus parameters, but also by taxing the attention and processing load with distractors, to which children may be particularly susceptible. Furthermore, adult levels of violations of the race model in distracting conditions may only be attained within the period from 15 to 19 years of age, in conjunction with the rapid maturation of “executive functioning,” including processes of working memory, selective attention and response inhibition, during this time-frame (Travis, 1998; Conklin et al., 2007; Couperus, 2011). Indeed there is a great deal of behavioral, physiological and anatomical literature demonstrating immature cortical frontal and parietal attention systems in children (e.g., Crewther et al., 1999; Konrad et al., 2005; Braddick and Atkinson, 2011; Klaver et al., 2011), as well as ongoing myelination which underpins cognitive development in the period from late childhood through mid to late adolescence (e.g., Travis, 1998; Dehaene-Lambertz et al., 2002; Barnea-Goraly, 2005; Paus, 2005; Klaver et al., 2011).

Recent literature from Otto and Mamassian (2012) has highlighted potential inherent limitations in the analysis of multisensory facilitation via the test of the race model inequality. The authors have suggested that multisensory facilitation and violations of the race model assumptions could be accounted for by increases in the variability of RTs in response to audiovisual signal processing, rather than integration between the sensory systems (Otto and Mamassian, 2012). In the present study, RT variability did not increase for multisensory targets, and in some cases a decrease in variability was observed, for multisensory compared to unisensory signal responses. In addition, trial history has been suggested to be a source of increases in variability (Otto and Mamassian, 2012; and see Spence et al., 2001). However, although this is an important area of research, a full assessment of trial history is beyond the scope of this study. It is important to note that in the current study, stimuli were presented randomly with equal probability, therefore, both unisensory and multisensory stimuli are equally likely to be affected by trial history. Overall, given that RT variability did not increase for multisensory target signals in the present study (see Figures 5, 6), changes in variability are unlikely to explain violations of the race model in this case. Differences in findings between studies may be due to the fact that only well learnt and ecologically congruent (i.e., matching) multisensory targets were used here while in previous studies looming visual stimuli and static sound, which are incongruent by nature, were used (Otto and Mamassian, 2012). Thus, differences in findings may be related to the differences in prior experience and learned associations with the stimuli used to assess multisensory facilitation across the studies (e.g., Vatakis and Spence, 2007; Thomas et al., 2010). Indeed, experience may shape the development of bottom-up and top-down multisensory processes, as demonstrated in various animal models (e.g., King et al., 1988; Wallace et al., 2006). Thus, further research is required to investigate what multisensory processes (e.g., parallel, interactive or integrative) are driving the facilitation observed in the current study.

## Conclusion

The present study expands current understanding of the effects of unisensory and audiovisual distractors on multisensory processing of objects and shows that, in general, these processes follow a consistent pattern across development. Children, adolescents and adults demonstrated multisensory facilitation of RTs in all distractor conditions but fewer violations of the race model were recorded in the presence of competing visual and audiovisual distractor objects. Multisensory facilitation could not be explained by changes in RT variability, suggesting that tests of race model violations may still have theoretical value at least for familiar multisensory stimuli.

Nonetheless, developmental improvements are observed from childhood to adolescence, and children and adolescents show a greater level of sensitivity to uni- and multisensory distractors compared to adults. The patterns of results seen here are likely driven by a complex interplay between developmental and specific distractor modality effects, for which, the relative contributions of bottom-up and top-down processing (including perceptual, attention and other higher order cognitive processes) require investigation. Even at age 15 years, aspects of multisensory processing are immature in distracting conditions. This line of research is important because the results may have implications for communication practices within educational and occupational settings, highlighting the benefits of congruent audiovisual stimuli in capturing attention and in reducing the effects of common environmental distractions. This may be particularly important for developing new strategies to minimize the impact of distractors in complex school or clinical environments, particularly benefiting those with cognitive or developmental disorders such as intellectual disability or attention deficit disorder.

## Author contributions

Conception and Design Harriet C. Downing, Sheila G. Crewther and Ayla Barutchu, data collection Harriet C. Downing, analysis Harriet C. Downing, Ayla Barutchu and Sheila G. Crewther, first draft of manuscript Harriet C. Downing, final edit and proof Harriet C. Downing, Sheila G. Crewther and Ayla Barutchu. All authors agree to be accountable for all aspects of the work ensuring that all questions related to the accuracy or integrity of any part of the work are appropriately investigated and resolved.

### Conflict of interest statement

La Trobe University Ethics Approval Number UHEC 08-139. Catholic Education Office Ethics Approval Number GE10/0009. The authors declare that the research was conducted in the absence of any commercial or financial relationships that could be construed as a potential conflict of interest.
